# A Rare Case of SARS-CoV-2-Induced Microscopic Polyangiitis

**DOI:** 10.7759/cureus.15259

**Published:** 2021-05-26

**Authors:** Nishant Allena, Jay Patel, Georgette Nader, Madanmohan Patel, Boris Medvedovsky

**Affiliations:** 1 Internal Medicine, BronxCare Health System, Bronx, USA; 2 Nephrology, BronxCare Health System, Bronx, USA

**Keywords:** covid 19, anca associated vasculitis, glomerulonephritis (gn), diffuse alveolar hemorrhage, microscopic polyangiitis, rituximab, acute kidney injury

## Abstract

Severe acute respiratory syndrome coronavirus 2 (SARS-CoV-2) infection triggers elevated levels of circulating cytokines and immune-cell hyperactivation, called a cytokine storm, which leads to dysregulated immune response not only towards the pathogen itself but also contributes to cellular, vascular injury and multiorgan dysfunction. The cytokine-induced endothelial inflammation and vascular pathology of COVID-19 is well reported in post-mortem biopsies and several cases reporting small, medium and large vessel micro/macro thrombotic events and vasculitis in multiple organs. So far, few cases have been reported with newly diagnosed antineutrophil cytoplasmic antibodies (ANCA)-associated vasculitis at the time of acute COVID-19 infection. The exact pathophysiology of SARS-CoV-2 and ANCA-associated vasculitis continues to be studied and reviewed.

Here we report a case of a 60-year-old female who presented to our institution with sudden onset of shortness of breath and hemoptysis. A detailed history revealed a recent severe acute respiratory syndrome coronavirus 2 (SARS-CoV-2) infection. Labs showed elevated serum creatinine, urine analysis with large blood and nephrotic range proteinuria. CT chest was remarkable for abnormal appearance of the parenchyma bilaterally compatible with a crazy paving pattern, suggesting pulmonary alveolar proteinosis versus diffuse alveolar hemorrhage. Vasculitis was suspected and the patient was started on IV corticosteroids and plasmapheresis. Diagnostic workup was positive for antineutrophil cytoplasmic antibodies-myeloperoxidase (ANCA-MPO), anti-Sjögren's syndrome-related antigen A autoantibodies (anti-SS-A) and antinuclear antibodies (ANA). Renal biopsy confirmed focal segmental necrotizing, crescentic and sclerosing glomerulonephritis, pauci-immune type, anti-MPO antibody/P-ANCA associated. A diagnosis of microscopic polyangiitis was made and she was started on rituximab immunosuppressive therapy following which she showed clinical improvement.

In this document, we present a unique case of microscopic polyangiitis possibly induced by SARS-CoV-2 infection confirmed by renal biopsy and clinical presentation. In the current setting of a global pandemic, we strongly recommend that vasculitis be high on the differential diagnosis in patients who are currently infected or had been infected with SARS-CoV-2 and present with acute kidney injury (AKI).

## Introduction

Vasculitis occurs when blood vessels become inflamed, causing them to thin and form aneurysms or narrow leading to organ ischemia [1]. Any size and type of blood vessel can become inflamed, resulting in varying presentations that predominantly affect a population subset. Immunofluorescent staining can be used to aid in diagnosis and differentiate between the subtypes.

Antineutrophil cytoplasmic antibodies (ANCA) directed against neutrophil serine protease proteinase 3 (cANCA) and perinuclear immunofluorescence pattern (pANCA) contribute to the pathogenicity of disease and are used in conjunction with clinical history and biopsy to diagnose vasculitis [1].

Vasculitis is classified as primary when there is no identifiable cause of vessel wall inflammation. A vasculitis is defined as secondary when blood vessel inflammation is a result of underlying disease or exposure to medications and/or infections like hepatitis B and tuberculosis [2]. Recent studies have shown that the novel coronavirus disease (COVID-19) has the potential to induce secondary vasculitis in patients [3,4]. Pathogenesis surrounding COVID-19-induced secondary vasculitis is still uncertain, however some researchers have hypothesized a cross-reactivity between SARS-CoV-2 antigen and autoantibodies in autoimmune disease [5]. Understanding the relationship between SARS-CoV-2 and autoimmunity is crucial in the diagnosis of vasculitis and treatment of the disease.

## Case presentation

A 60-year-old female was admitted to the hospital with a sudden onset of severe epigastric pain and coughing up blood-tinged sputum. She had a remarkable medical history of hypertrophic obstructive cardiomyopathy, coronary artery disease, asthma, hypertension, hyperlipidemia and history of tobacco use for 15 years. The patient reported no prior history of any connective tissue disorder, kidney disease, allergies, alcohol, illicit drugs or recent travel history. She also revealed that she tested positive for COVID-19 twice, in April and December, 2020 and presented to us in January, 2021. In the Emergency Department, she was observed to be hyperventilating but without apparent distress. Her vital signs were significant for an elevated BP of 165/84 mmHg, pulse of 76 beats per minute, respiratory rate of 19 breaths per minute and a temperature of 98.4 F. Physical examination at the time of presentation was significant for epigastric tenderness on palpation and bibasilar crackles on auscultation.

**Figure 1:**
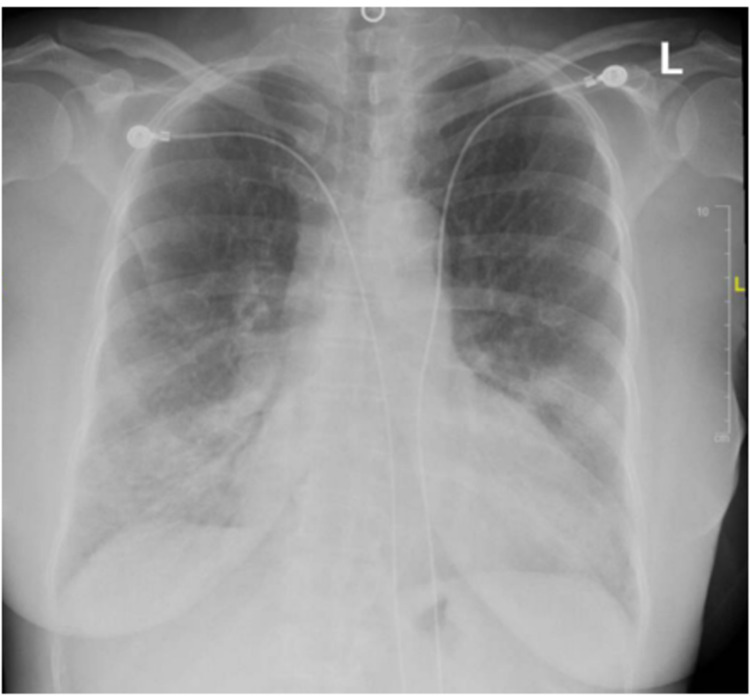
Chest X-ray showing bilateral patchy infiltrates

Laboratory testing on presentation reported a leukocyte count of 11.0 k/ul, hemoglobin of 9 g/L, platelet counts of 394,000/mm^3^, an erythrocyte sedimentation rate of 120 mm/hr, C-reactive protein of 28.43 mg/L (ref <5.0), serum creatinine of 4.3 mg/dL and a lipase of 300 U/L. Urinalysis showed large blood and nephrotic range proteinuria (Urine protein to creatinine ratio of 5.6 g/day), SARS-CoV-2 antibodies were positive. A chest X-ray showed bilateral patchy infiltrates (Figure 1).

**Figure 2:**
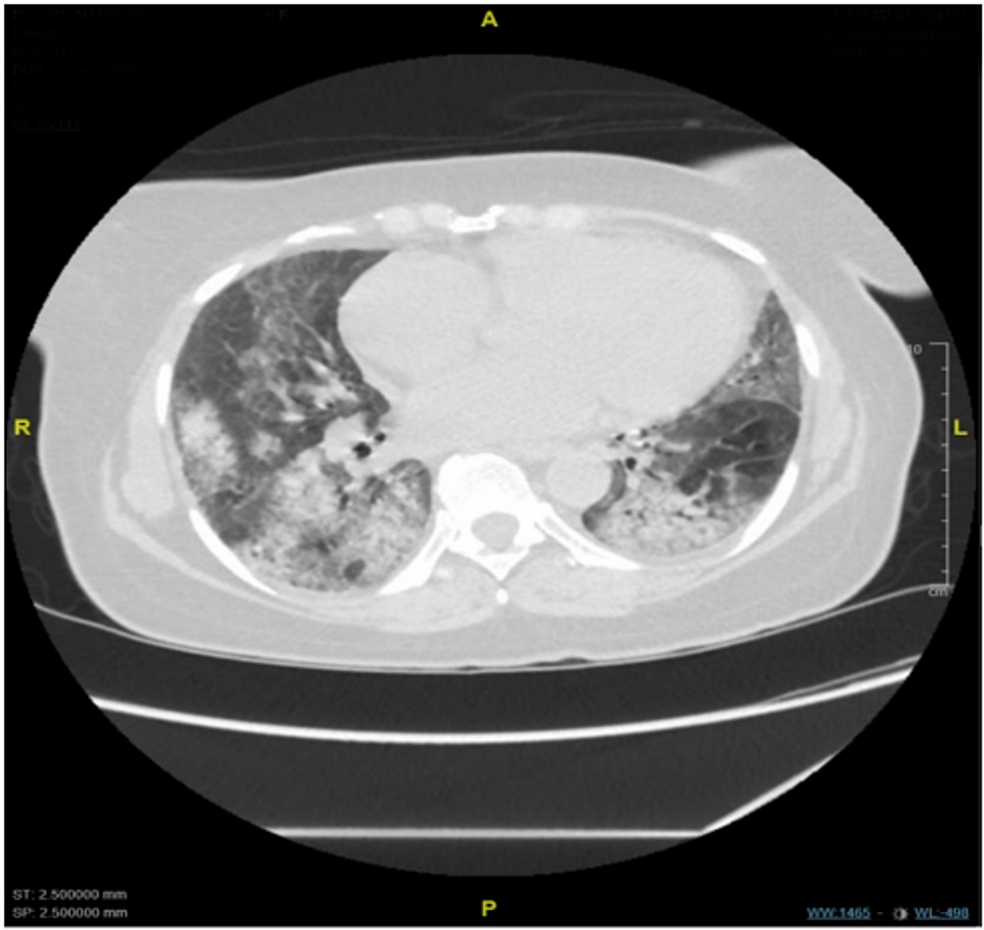
CT chest without contrast showing an abnormal appearance of the parenchyma bilaterally compatible with a crazy paving pattern

CT chest reported an abnormal appearance of the parenchyma bilaterally compatible with a crazy paving pattern (Figure 2).

In the setting of the current presentation, vasculitis was suspected and a full glomerular workup was ordered. The patient was seen by Gastroenterology for pancreatitis and hydration was recommended with esophagogastroduodenoscopy as outpatient.

A day into the admission the patient’s hemoglobin dropped to 7.8 g/dL and further trended down to 6.7 g/dL on day 3 of admission warranting a blood transfusion. Nephrology was consulted for elevated creatinine level of 4.3 and nephrotic range proteinuria and a decision was made to start the patient on pulse steroids and subsequently started on 1000 mg of intravenous methylprednisolone. After the second dose of methylprednisolone, an improvement in serum creatinine was noted. A bronchoscopy was planned for the evaluation of suspected alveolar hemorrhage. Prior to undergoing bronchoscopy, the patient became hypoxic and required oxygen supplementation via nasal cannula. She further desaturated while undergoing a blood transfusion which was stopped for suspicion of transfusion-related acute lung injury (TRALI)/transfusion-associated circulatory overload (TACO). She was then placed on bilevel positive airway pressure (BiPAP) and transferred to the ICU.

In the setting of high suspicion of alveolar hemorrhage, an acute drop in hemoglobin and worsening hypoxia, the patient was initiated on plasmapheresis. A total of five sessions of plasmapheresis were carried out subsequently after which the patient was put on maintenance steroids of 60 mg prednisone daily. Antineutrophil cytoplasmic antibodies-myeloperoxidase (ANCA-MPO) (>800), anti-SS-A and antinuclear antibodies (ANA) (Titer 1:1280, cytoplasmic pattern) came back positive. Rituximab was administered as a part of induction therapy. Her oxygen requirement also decreased and was weaned to a nasal cannula and finally onto room air.

**Figure 3:**
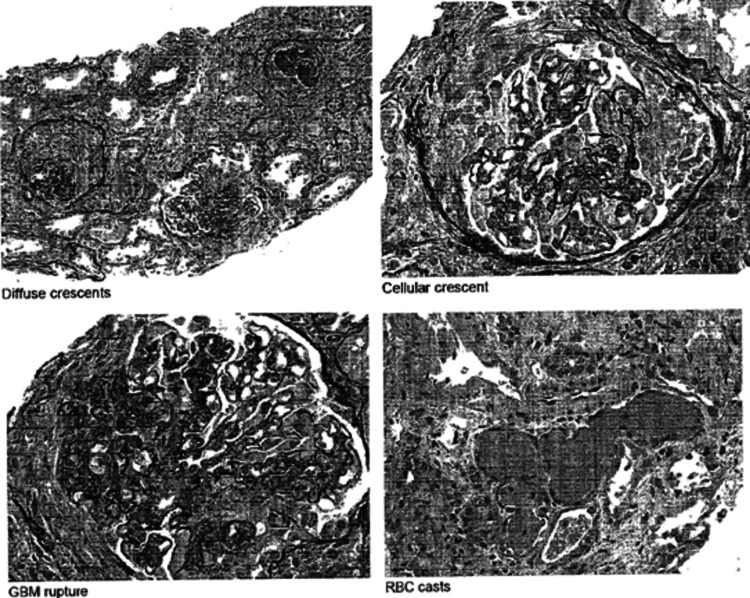
Histopathology from biopsy

A renal biopsy was done to confirm the diagnosis and biopsy results reported focal segmental necrotizing, crescentic and sclerosing glomerulonephritis, acute and chronic, severe, pauci-immune type (anti-MPO antibody/P-ANCA associated) (Figure 3).

It also revealed tubular atrophy and interstitial fibrosis, moderate (40%) with moderate interstitial inflammation, acute tubular injury and focal blood cell casts along with arterio and arteriosclerosis, mild to moderate confirming our initial diagnosis.

The patient’s creatinine improved to 2.4 mg/dL and she was discharged on maintenance steroids and scheduled for the second dose of rituximab two weeks from the date of the initial dose.

## Discussion

Microscopic polyangiitis (MPA) is a form of ANCA vasculitis. ANCA vasculitis is caused by host-derived auto-antibodies against shielded neutrophilic antigens. These antibodies are suspected to react against granules present in neutrophils and monocytes. 70% of MPA cases have a positive ANCA at the time of diagnosis [6]. The annual incidence of ANCA vasculitis is estimated to be 3.3 per 100,000 with a prevalence of 42.1 per 100,000. The annual incidence of MPA was estimated to be 1.6 per 100,000 [7]. MPA has a slightly male predominance, a ratio of 1.8:1, and an average age of onset between 50-60 years [8]. MPA has a variety of manifestations affecting many different organs; however most cases of MPA have been associated with renal and lung involvement. The presentation has also varied from patient to patient, with the most common being flu-like symptoms and arthralgia [8]. The most common presentation in MPA is usually a renal manifestation and is estimated to be seen in 80-100% of patients [8]. Most patients present with a rapidly progressive glomerulonephritis picture, which presents as a loss of renal function in days to weeks, a urine analysis that has protein and red blood cells, and histological findings on biopsy that shows crescent cellular formation on glomeruli [9]. Histologically MPA shows a necrotizing small vessel vasculitis with little or absent immune deposits, pauci-immune vasculitis [10]. MPA is treated with immunosuppressive therapy. Patients with organ threatening MPA are started on glucocorticoids with either cyclophosphamide or rituximab [11]. Plasmapheresis has also been known to help those patients that have severe kidney disease, a creatinine > 4.0, and active vasculitis [11].

In our case, we have not deviated from the usual guidelines of treatment for ANCA vasculitis following which the patient has shown clinical improvement. At this point in time, we do not have many studies regarding the long-term effects of COVID-19-induced MPA and from our experience, the clinical prognosis followed the same course as regular MPA, though more studies are required in this field to analyze its effects over the long term.

COVID-19 is a viral pandemic that has not been fully understood. There are over 132 million cases of COVID reported all over the world as of April 6th, 2021 [12]. The virus is known to cause acute respiratory distress syndrome, however, there are some speculations that the virus could be a direct invader of endothelial cells, and may cause vasculitis [13]. The incidence of COVID-19 in patients with ANCA-associated vasculitis appears to be similar to that of the general population [14]. There has been another case of a patient with COVID-19-associated microscopic polyangiitis reported as well [15]. Uppal et al. have also reported cases with de novo ANCA vasculitis with glomerulonephritis in patients with COVID-19 [16].

With a possibility of COVID-19-associated vasculitis, there should be a higher level of suspicion of vasculitis in patients diagnosed with COVID-19 and presenting with acute kidney injury in the proper clinical setting.

## Conclusions

With COVID-19 being a relatively new disease, its effects on various organ systems have not been fully understood. Initial reports indicated a Kawasaki-like disease predominantly in younger patients and cases of cutaneous vasculitis in adults with COVID-19 have also been reported. In this document, we have presented a unique case of microscopic polyangiitis possibly induced by SARS-CoV-2 infection confirmed by renal biopsy and clinical presentation. In the current setting of a global pandemic, we strongly recommend that vasculitis be high on the differential diagnosis in patients who are currently infected or had been infected with SARS-CoV-2 and present with acute kidney injury.
